# Reduced breakthrough symptom exacerbations in patients with biochemically controlled acromegaly switched from injected depot somatostatin receptor ligands to once-daily oral paltusotine in the PATHFNDR-1 clinical trial

**DOI:** 10.1007/s11102-026-01733-2

**Published:** 2026-07-23

**Authors:** David R. Clemmons, Tiffany P. Quock, Alessandra Casagrande, Yang Wang, Alan Krasner

**Affiliations:** 1 Department of Medicine, University of North Carolina at Chapel Hill, Chapel Hill, NC USA; 2 Crinetics Pharmaceuticals, Inc, San Diego, CA USA

**Keywords:** Acromegaly, Insulin-like growth factor 1, Patient-reported outcomes, Somatostatin/analogs and derivatives, Symptom exacerbation, Symptom evaluation

## Abstract

**Purpose:**

To evaluate the hypothesis that breakthrough acromegaly symptom exacerbation frequency would be reduced after switching biochemically controlled patients from depot somatostatin receptor ligand (SRL) injections to once-daily oral paltusotine.

**Methods:**

Biochemical disease control (insulin-like growth factor 1 [IGF-I]) and Acromegaly Symptom Diary (ASD) data were analyzed from PATHFNDR-1, a 36-week, randomized, placebo-controlled, double-blind, phase 3 trial of paltusotine in patients with acromegaly biochemically controlled (IGF-I ≤ 1.0× ULN) with SRLs. The ASD measured the daily severity of 7 core and 2 exploratory acromegaly symptoms, each rated on a scale from 0 to 10 based on 24-hour recall. Breakthrough acromegaly symptom exacerbations were defined as ≥ 2-point increases for any individual symptom score, comparing a 2-day average with the prior 2-day average.

**Results:**

Despite no significant changes in IGF-I levels or overall core symptom severity scores, mean (SE) symptom exacerbation frequencies declined progressively after switching to paltusotine, from 30.2% (5.6%) of days during screening (SRL treatment) to 6.2% (1.6%) of days (*n* = 22, *p* < 0.0001). The reduction in individual symptom exacerbation frequencies with paltusotine treatment was consistent across all acromegaly symptoms assessed. IGF-I levels did not correlate significantly with symptom severity scores or symptom variability.

**Conclusion:**

In patients with acromegaly biochemically controlled with injected SRLs, switching to once-daily oral paltusotine was associated with stable biochemical control, stable symptom severity, and a significantly reduced frequency of symptom exacerbations. A simple daily symptom assessment tool provided important information pertaining to acromegaly disease control that was not apparent from IGF-I measurements.

**Supplementary Information:**

The online version contains supplementary material available at https://doi.org/10.1007/s11102-026-01733-2.

## Introduction

Acromegaly is a rare disease primarily caused by a pituitary adenoma that secretes excess growth hormone (GH), leading to elevated levels of insulin-like growth factor 1 (IGF-I) [1, 2]. Medical therapy is indicated in patients with persistent GH hypersecretion following surgical resection of the pituitary tumor, or in those for whom surgery is contraindicated [2–4]. Historically, the most common medical therapies for acromegaly have been injected depot somatostatin receptor ligands (SRLs) [1, 2].

Knowledge about the control of acromegaly symptoms achieved with depot SRLs is limited [5]. Even less is known about the symptom variability associated with depot SRL treatment. The literature is clear that patients on these medications, even if biochemically controlled, frequently report breakthrough symptom exacerbations, sometimes occurring at random times and some in identifiable patterns (e.g., toward the end of the injection cycle) [6–8]. Consistent with these findings, rising IGF-I levels late in the injection cycle have been described, even in patients considered to have achieved biochemical control [9, 10]. These observations underscore an unmet need to reduce the variability of disease control in medically treated patients with acromegaly, including the need to reduce the frequency of breakthrough symptom exacerbations, even in those patients who appear to have IGF-I levels within normal limits.

Paltusotine is a nonpeptide, highly selective somatostatin 2 (SST2) receptor agonist that has high oral bioavailability, reaches steady-state drug exposure within 1 week, and delivers consistent drug effects with once-daily oral administration [11–13]. Paltusotine is approved by the US Food and Drug Administration (FDA) for the treatment of adults with acromegaly who had an inadequate response to surgery and/or for whom surgery is not an option [14]; additionally, it is approved in Europe for the treatment of adult patients with acromegaly [15].

To evaluate the effects of paltusotine on acromegaly symptoms, the Acromegaly Symptom Diary (ASD) [16] was developed in accordance with FDA guidance for use in clinical trials, and the symptoms identified for inclusion in the ASD were previously shown to be important to patients with acromegaly [16]. Because the ASD is completed daily, it allows for quantitative assessment of day-to-day symptom variability. The acromegaly symptom exacerbation frequency (ASEF) calculated using this daily symptom assessment tool has recently been shown to correlate with multiple aspects of reported disease burden, including impaired daily activities, reduced work productivity, reduced treatment satisfaction, diminished life satisfaction, and increased healthcare resource utilization (see companion article: Geer EB, et al. “Symptom Severity and Exacerbation Frequency in Medically Treated Patients With Acromegaly”).

The phase 3 PATHFNDR-1 study (NCT04837040) was a double-blind, placebo-controlled clinical trial designed to evaluate the efficacy and safety of paltusotine compared with placebo in patients biochemically controlled on depot octreotide or lanreotide [17]. The total core symptom severity score, measured using the ASD, was statistically superior in patients who switched from injections to paltusotine versus placebo, as previously reported [17].

Because PATHFNDR-1 demonstrated maintenance of baseline normal IGF-I levels during treatment with paltusotine [17], this study affords the opportunity to evaluate changes in symptom variability without the potential confounders of changing biochemical control or changing symptom severity. Here, we explore the hypothesis that daily oral administration of paltusotine would be associated with a reduction in the frequency of breakthrough symptom exacerbations when switching from injectable depot SRLs in biochemically controlled patients with acromegaly.

## Methods

### PATHFNDR-1 design and patients

The methodological details of PATHFNDR-1 have been previously reported [17]. Briefly, PATHFNDR-1 was a phase 3, multinational, randomized, double-blind, placebo-controlled trial that included a screening period (during which patients received their last SRL injection), a 36-week period of randomized treatment, and an open-label extension phase (Online Resource: Suppl. Figure 1). Eligible patients were at least 18 years of age, had a confirmed diagnosis of acromegaly, and were biochemically controlled (IGF-I ≤ 1.0× upper limit of normal [ULN] based on the average of 2–3 separate measurements at study entry) on a stable dose (≥ 12 weeks before study entry) of injectable depot lanreotide or octreotide. Key exclusion criteria were pituitary surgery within 24 weeks of screening; a history of pituitary radiation; use of pegvisomant, cabergoline, or short-acting SRLs within 12 weeks or pasireotide within 24 weeks; and poorly controlled diabetes or cardiovascular, renal, or hepatic disease.

Enrolled patients were randomly assigned (1:1) to paltusotine or placebo and stratified based on IGF-I level (< 0.86× ULN or ≥ 0.86× ULN) and prior depot SRL (octreotide or lanreotide) using a fixed-block randomization scheme. Paltusotine (or matching placebo) was initiated at a starting dose of 40 mg/day and was titrated to 60 mg/day during the first 24 weeks of the randomized treatment period if IGF-I was > 0.9× ULN and tolerability was acceptable. At any time, paltusotine could be down-titrated (to a minimum of 20 mg/day) based on tolerability. Rescue medication consisting of the patient’s prior depot SRL was to be administered if 2 consecutive IGF-I levels were ≥ 1.3× ULN at the highest dose of study medication and the patient experienced new acromegaly symptoms or an exacerbation of existing symptoms per investigator assessment. Rescued patients were encouraged to remain in the study after restarting depot SRL injections (and no longer receiving study drug) for the remainder of the randomized controlled period.

The primary endpoint in PATHFNDR-1 was the proportion of patients with a mean IGF-I ≤ 1.0× ULN during weeks 34 and 36 of the randomized treatment period. Serum IGF-I and GH (assessed at 5 time points at least 30 min apart within a 3-hour period) were measured in a central laboratory using iSYS immunoassays (Immunodiagnostic Systems).

Beginning in the screening period and throughout the randomized controlled period, acromegaly symptoms were reported using the daily ASD. This instrument includes 7 core acromegaly symptoms (headache, joint pain, sweating, fatigue, leg weakness, swelling, numbness/tingling) plus 2 additional symptoms (sleep difficulty and short-term memory difficulty), each rated based on 24-hour recall on a scale from 0 (no symptom) to 10 (worst symptom) [16]. The ASD total core score was derived by summing the scores for the 7 core symptoms (range, 0–70).

### Statistical analysis

Change from baseline in mean IGF-I and ASD total score were summarized by visit for all patients randomized in PATHFNDR-1. A last observation carried forward (LOCF) approach was used to account for missing data for patients who received rescue medication or discontinued from the study. Coefficients of variation (CV) for IGF-I levels during stable dosing with paltusotine or placebo were calculated for each repeated measure for each patient based on this formula: CV = [(IGF-I SD)_n_/(IGF-I average)_n_]%, with n indicating sample size.

A separate qualitative study examined the patient perspective of meaningful change on the ASD. Trained qualitative researchers conducted one-on-one interviews, and a change of ≥ 2 points on individual ASD items was identified by patients with acromegaly as clinically meaningful (data on file). Breakthrough symptom variability was quantified as the ASEF, defined as the percent of days with at least a 2-point increase in any 1 or more of the 9 acromegaly symptoms scores, based on comparison of a 2-day average (of day x and day x + 1) to the prior 2-day average (of day x-1 and day x-2).

A post hoc analysis of ASEF trends over time was performed in participants who had adequate ASD data both during the screening period while treated with injected SRLs and after switching to paltusotine (up to 9 months). Of 30 participants randomized to paltusotine, those who completed ≤ 3 days of ASD data during the screening period (*n* = 6) and those who could not be evaluated during the stable dose period of the study (months 6–9; because of prior rescue medication initiation; *n* = 2) were excluded from the analysis. A sensitivity analysis for the definition of ASEF was conducted using time windows of 1, 3, and 5 days (primary analysis: 2-day window).

A linear regression model controlling for the difference in baseline breakthrough symptom exacerbation frequency was used to evaluate treatment group differences in ASEF. Of 58 total participants randomized into the study, 12 who had ≤ 3 days of ASD completion during the screening period were excluded from this analysis. Symptom data following receipt of rescue medication were excluded.

Spearman correlation coefficients and associated nominal *p* values were calculated for associations of total symptom severity (total ASD) scores at screening and at the end of treatment with ASEF and for associations of IGF-I levels and GH levels with total symptom severity scores and ASEF.

## Results

### Patients and rescue medication use

As previously reported [17], 58 patients were enrolled in PATHFNDR-1 and received at least 1 dose of study medication (paltusotine, *n* = 30; placebo, *n* = 28). Rescue medication (injected SRL) was received by 17 patients in the placebo group (60.7%) and 2 patients in the paltusotine group (6.7%) during the randomized treatment period (*p* < 0.0001) [17]. All enrolled patients were required to complete the ASD, beginning in the screening period during depot SRL treatment. The mean (SD) number of days with ASD data was 17.2 (14.7) during the screening period; in the randomized treatment period (prior to administration of rescue medication), these values were 188.7 (51.2) in the paltusotine group and 141.5 (75.7) in the placebo group. The mean completion rate of daily ASD questionnaires was 67.0% during screening, 76.8% during randomized treatment with paltusotine, and 82.9% during randomized treatment with placebo. The study discontinuation rate was 1.7% [17].

### Biochemical control and variability

**Fig. 1:**
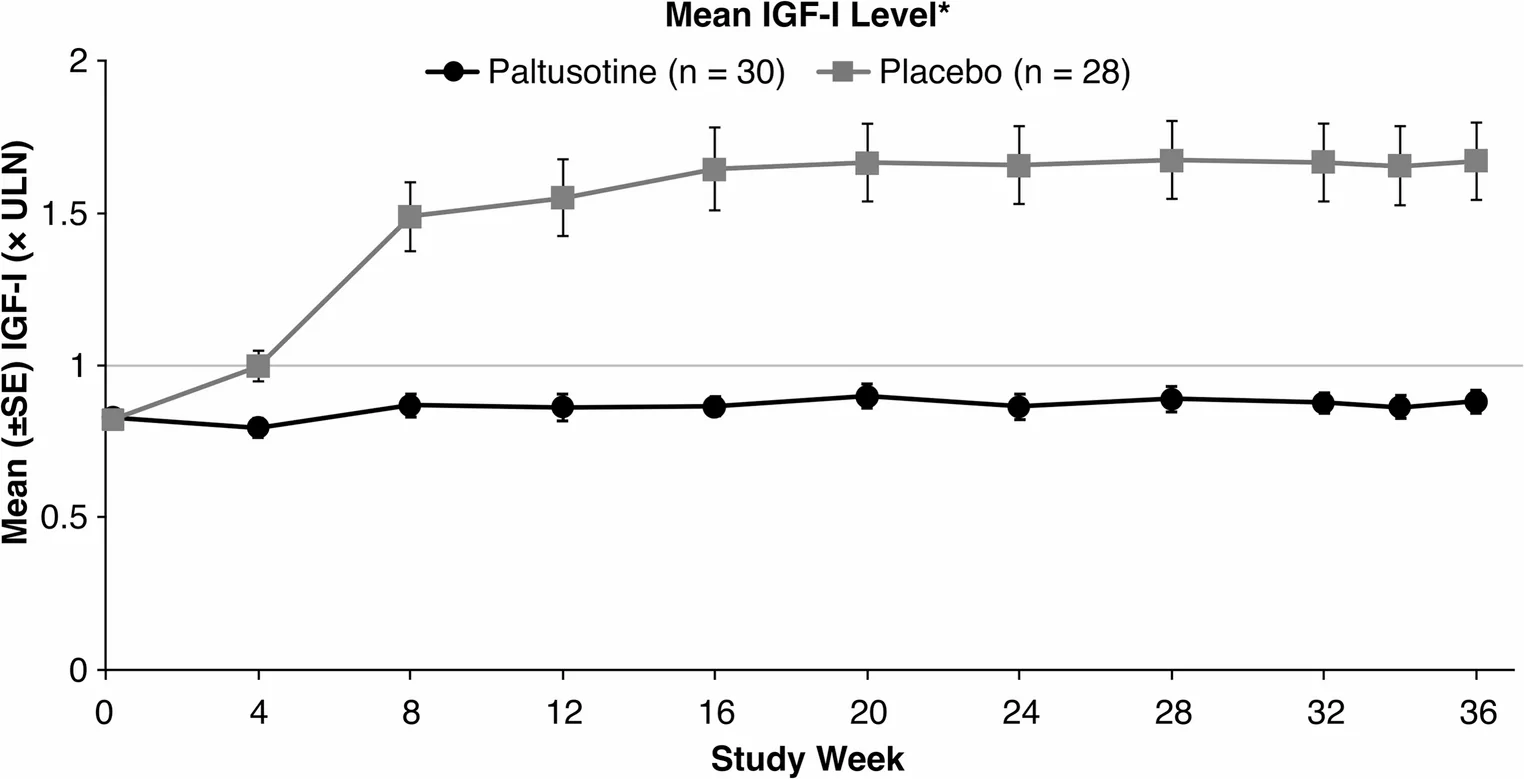
Mean (SE) change from baseline in IGF-I (× ULN) by study week for patients receiving randomized treatment at each time point in PATHFNDR-1. *LOCF for patients who received rescue medication or discontinued from the study. IGF-I, insulin-like growth factor 1; LOCF, last observation carried forward; ULN, upper limit of normal

In PATHFNDR-1, mean IGF-I increased in the placebo group after switching from depot SRL therapy and remained stable throughout the randomized treatment period in the paltusotine group, with separation between paltusotine and placebo observed by week 8 (Fig. 1). At the end of randomized treatment, mean IGF-I had increased from 0.82× ULN to 1.68× ULN with placebo and was relatively unchanged (0.83× ULN to 0.89× ULN) with paltusotine [17]. The CV (SD) of IGF-I levels during stable dosing (excluding data after rescue medication was administered) was 10.8% (6.0%) for paltusotine and 27.5% (15.0%) for placebo.

### Symptom severity and variability

**Fig. 2:**
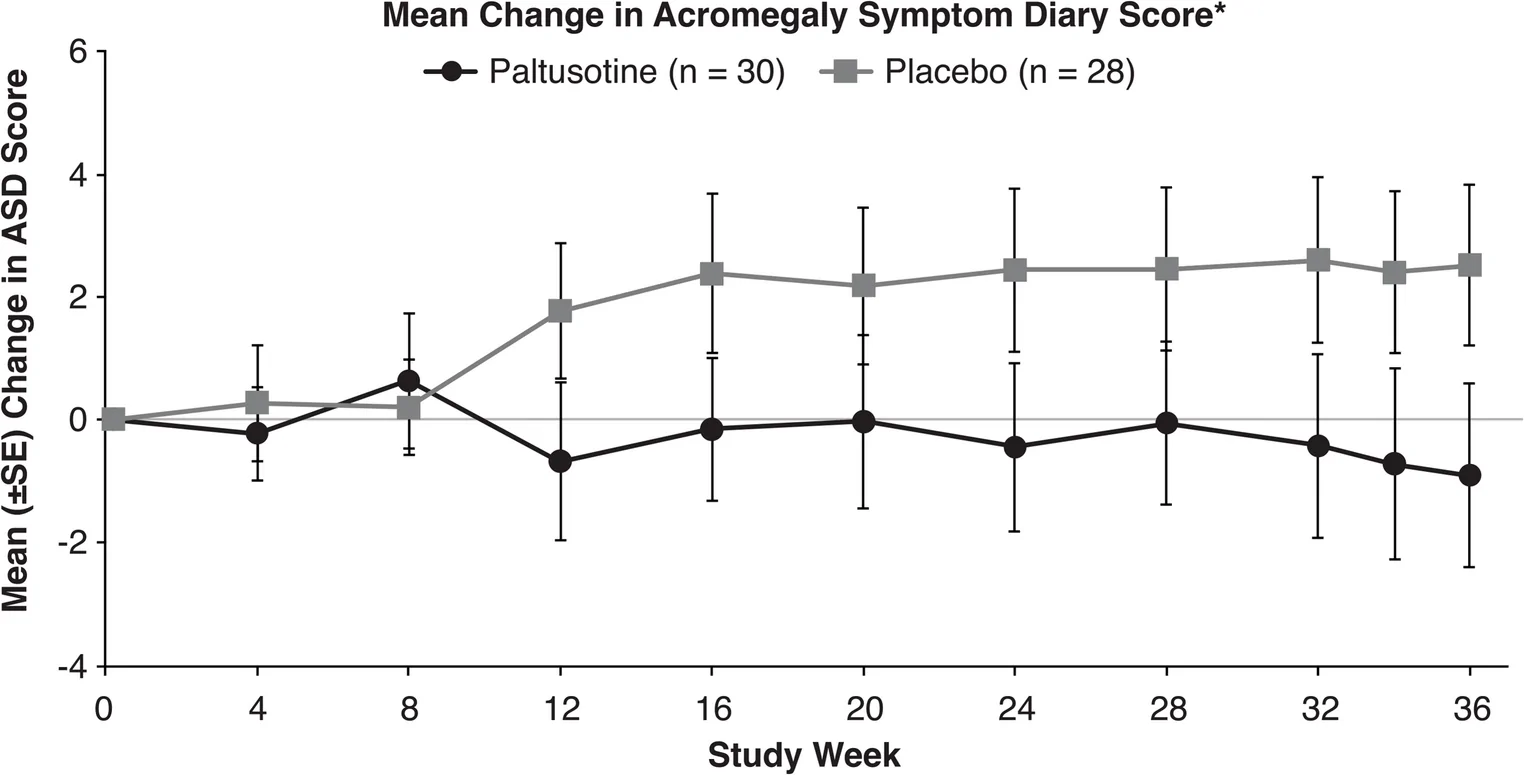
Mean (SE) change from baseline in ASD total severity score by study week for patients receiving randomized treatment at each time point in PATHFNDR-1. *LOCF for patients who received rescue medication or discontinued from the study. ASD, Acromegaly Symptom Diary; LOCF, last observation carried forward

The mean ASD total core severity score in PATHFNDR-1 remained stable after patients were switched from depot SRLs to paltusotine and increased (worsened) in the placebo group, with separation between paltusotine and placebo observed by week 12 of the randomized treatment period (Fig. 2). Mean scores for individual ASD symptoms generally remained stable with paltusotine and increased with placebo [17].

In the overall study population, most patients reported multiple acromegaly symptoms during the 2 to 4 weeks of ASD data collection during injected SRL treatment in the PATHFNDR-1 screening period, with 51.0% of patients experiencing more than 5 different symptoms (defined as an individual symptom score of > 2).

**Fig. 3:**
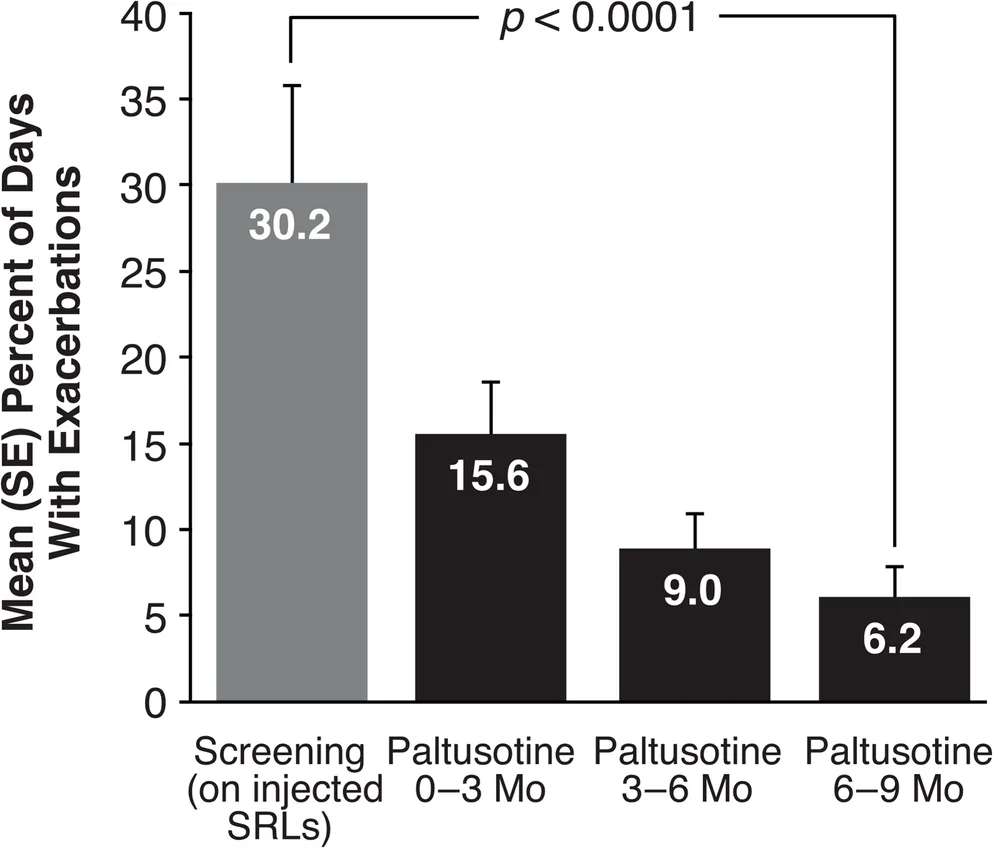
Acromegaly symptom exacerbation frequency in patients from PATHFNDR-1 who switched from injected SRLs to paltusotine. *P* value for the difference in symptom exacerbation frequency between screening and months 6 to 9 for the 22 patients in the paltusotine arm of PATHFNDR-1, based on a linear regression model. SRL, somatostatin receptor ligand

Post hoc analyses of symptom exacerbations included 22 patients from the paltusotine group. The mean symptom exacerbation rate was 30.2% of days during the PATHFNDR-1 screening period (Fig. 3), in which all patients had normal IGF-I levels while being treated with depot SRLs.

**Fig. 4:**
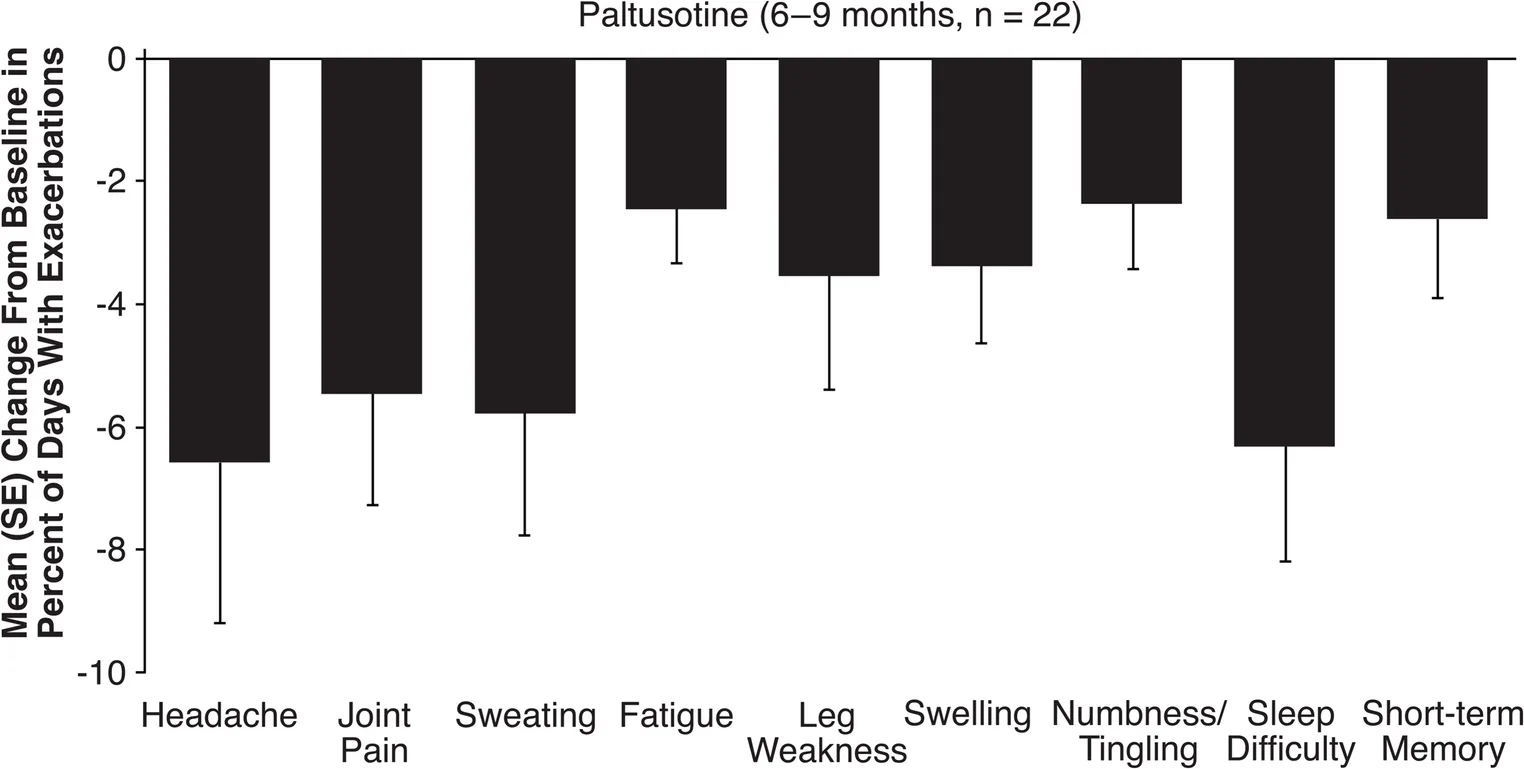
Change from injected SRL baseline in individual symptom exacerbation frequencies at 6 to 9 months in patients treated with paltusotine in PATHFNDR-1. SRL, somatostatin receptor ligand

In the context of stable biochemical control and symptom severity in paltusotine-treated patients in PATHFNDR-1, ASEFs declined progressively from a mean (SE) of 30.2% (5.6%) of days during treatment with depot SRLs in the baseline/screening period to 6.2% (1.6%) of days during stable dosing with paltusotine (*p* < 0.0001; Fig. 3). The change from baseline in ASEF during treatment with paltusotine was time-dependent, with initial reductions seen in the first 3 to 6 months, and further reductions observed in months 6 through 9, the latter of which was, per protocol, a stable dosing period. The reduction in individual symptom exacerbation frequencies with paltusotine treatment (at 6–9 months) was consistent across all acromegaly symptoms assessed (Fig. 4).

In a linear regression model controlling for baseline breakthrough symptom exacerbation frequency, paltusotine treatment was associated with significantly greater reduction in symptom exacerbation frequency compared with placebo (difference = -7.3%; *p* = 0.0167; *n* = 46).

### ASEF definition sensitivity analysis

**Fig. 5:**
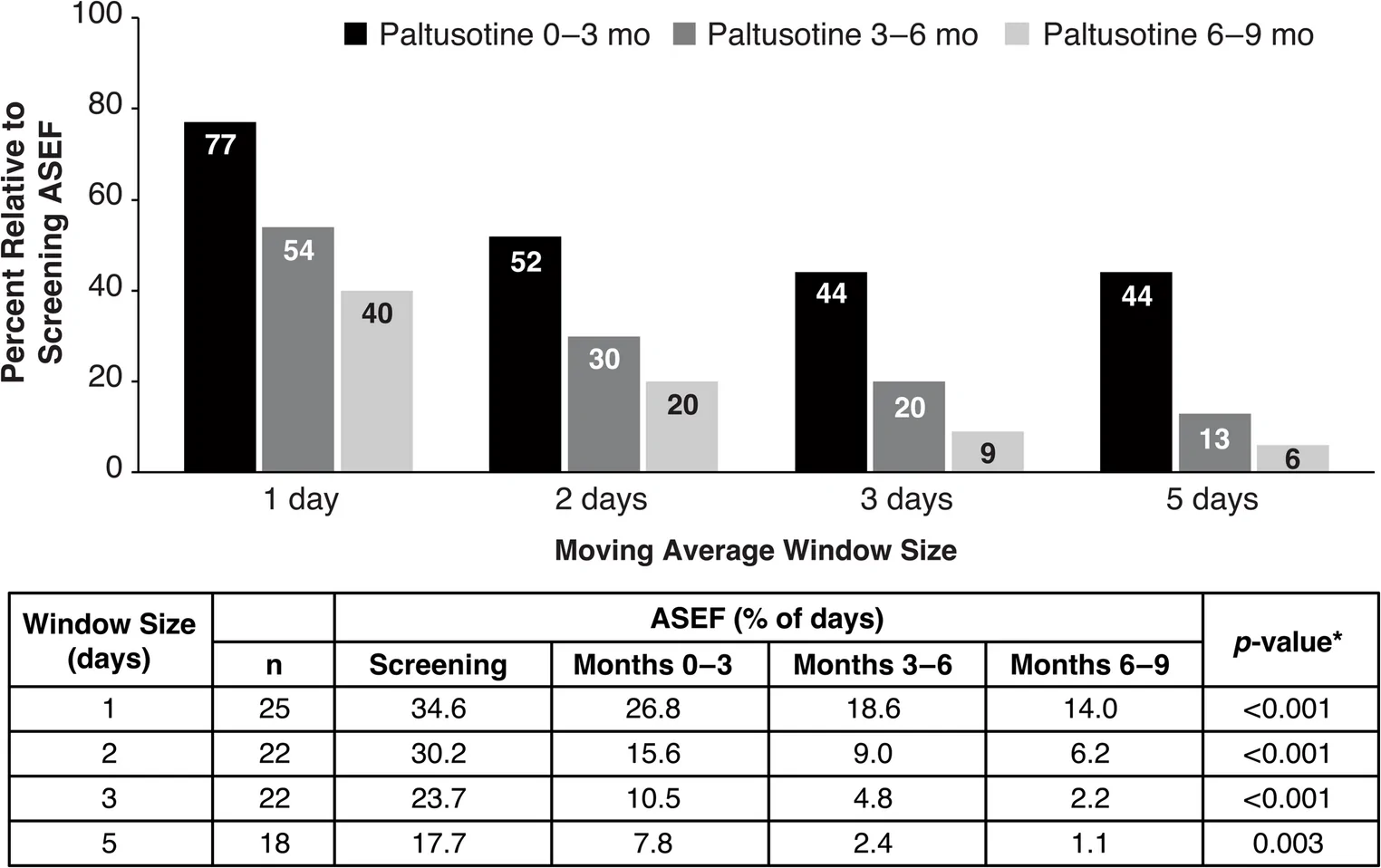
Sensitivity analysis of ASEF definition. Percent of days with acromegaly symptom exacerbation during treatment with paltusotine relative to injected depot SRLs for time windows of 1, 2, 3, and 5 days. Values shown are calculated as ASEF for paltusotine, divided by ASEF during screening, times 100. *Based on signed rank test comparing months 6 to 9 with screening. ASEF, acromegaly symptom exacerbation frequency; SRL, somatostatin receptor ligand

Sensitivity analyses assessed time windows of 1, 3, and 5 days to compute the ASEF. The results showed consistent directional trends across all evaluated time windows and were aligned with the results using the 2-day window (Fig. 5).

### Relationship between biochemical and symptom control

IGF-I levels did not correlate with total core symptom severity scores (*r* = 0.03, *p* = 0.838), or breakthrough symptom exacerbation rates at screening (ASEF: *r* = -0.03, *p* = 0.834) or during paltusotine treatment. Likewise, intra-day mean GH levels did not correlate with total symptom severity scores or measures of symptom variability.

## Discussion

In biochemically controlled patients with acromegaly who were switched from injected depot SRLs to once-daily oral paltusotine in PATHFNDR-1, control of both IGF-I levels and symptom severity was maintained; however, symptom variability was decreased. Mean breakthrough symptom exacerbation frequency was 2.1 days per week during screening while patients were treated with depot SRLs, similar to that previously observed in a separate study of outpatients receiving depot SRLs who completed daily symptom surveys for 90 consecutive days (see companion article: Geer EB, et al. “Symptom Severity and Exacerbation Frequency in Medically Treated Patients With Acromegaly”). In the present analysis, the frequency of breakthrough symptoms declined significantly from the depot SRL screening period, in a time-dependent manner, to 0.4 days per week during stable dosing (6–9 months) with paltusotine. Reductions were observed in exacerbation frequencies across all acromegaly symptoms assessed.

A mechanism for the reduced symptom variability during treatment with oral paltusotine compared with depot SRLs cannot be assessed from these data but could include irregular drug absorption patterns from deep parenteral administration. Octreotide long-acting release (LAR), approved for intramuscular administration, is associated with frequent inadvertent injections to the subcutaneous (SC) space, even when administered by experienced health care providers [18]. This can result in calcified radio-opaque SC nodules, which may interfere with drug absorption, in up to 50% of patients [18, 19]. Lanreotide depot (deep SC injection) is also associated with SC nodule formation [20]. These findings are consistent with results of a survey of 195 patients with acromegaly receiving depot SRLs, in which almost half of patients reported encountering problems with the preparation and administration of their SRLs, and more than 50% indicated that they had experienced acromegaly symptoms that were different between injections [6].

Although breakthrough symptoms are often reported to occur at the end of the injection cycle [6, 7, 10, 21], randomly dispersed breakthrough exacerbations in medically treated patients can be detected using a daily symptom assessment (see companion article: Geer EB, et al. “Symptom Severity and Exacerbation Frequency in Medically Treated Patients With Acromegaly”). The cause of unpredictable symptom exacerbations could reflect unmeasured day-to-day surges of GH. The current study did not measure GH frequently enough to evaluate this hypothesis. However, it is well described in the literature that breakthrough acromegaly symptoms (notably headache) can respond to as-needed bolus injections of short-acting octreotide used in addition to regularly administered depot SRL injections [22, 23].

To our knowledge, the frequency of breakthrough acromegaly symptom exacerbations has not been previously studied using prospectively collected daily symptom data. In this study, exacerbation was defined as a 2-point severity score increase for any symptom, over a moving 2-day average, compared with the average from the previous 2 days. Sensitivity analyses showed no change in the relative pattern of symptom exacerbation reduction over the study duration when evaluating time windows of 1 to 5 days for the calculation of the moving average. Two-day windows were selected as the primary analysis because (a) this minimizes exclusion of potentially clinically meaningful events without relying on unconfirmed single-day variations and (b) this definition of exacerbation (2-point increases in 2-day windows) has been shown to correlate with numerous measures of clinical meaningfulness (see companion article: Geer EB, et al. “Symptom Severity and Exacerbation Frequency in Medically Treated Patients With Acromegaly”).

When evaluating correlations between biochemical status and symptom control, one can find higher average symptom burden in biochemically uncontrolled populations [24]. However, data from this and other studies suggest that measurement of IGF-I alone does not correlate with symptom severity on an individual patient level, nor does it adequately capture the burden of breakthrough symptom exacerbations [6–8, 25]. In clinical practice, relying solely on biochemical information may contribute to the reported lack of alignment between patient perception and physician assessments of ongoing symptom burden [26]. Cursory assessments of symptom control that rely on long-term recall do not accurately capture symptom severity or variability (see companion article: Geer EB, et al. “Symptom Severity and Exacerbation Frequency in Medically Treated Patients With Acromegaly”). It is interesting to note that in this study, there was a time lag between IGF-I separation and symptom severity separation for paltusotine versus placebo. Some time might be required to equilibrate to a new biochemical milieu before any associated symptom changes are sensed and reported.

Findings from this analysis suggest that paltusotine reduced biochemical variability as well as symptom variability. The lack of consistent control of IGF-I observed in patients treated with a depot SRL is well documented [9, 10]. In PATHFNDR-1, daily oral paltusotine was associated with stable biochemical control over time. The fluctuation of IGF-I, expressed as CV of IGF-I, was 10.8 ± 6.0% in the paltusotine treatment group, which is comparable to that previously reported for patients with IGF-I results that remained consistently normal on medical treatment (11.6 ± 6.0%), and lower than results for the overall population treated with injected SRLs (14.4 ± 7.6%) [9].

The results of the present analyses may be limited with respect to generalizability because patients who enrolled in the study were willing to discontinue use of an injected depot SRL to try an experimental oral medication. As a result, the enrolled population may have been enriched with patients who were dissatisfied with the symptom control provided by depot SRLs. Other limitations of these analyses include the lack of a concurrent control group that maintained IGF-I control; therefore, the potential impact of time in study cannot be fully evaluated. The placebo group in this study does not serve as a suitable population to evaluate change in ASEF over time because of the loss of biochemical control, which resulted in the majority of patients meeting pre-specified protocol criteria for rescue during the randomized treatment period of the study. Only 11 of 28 (39%) patients randomized to placebo completed the 9-month treatment duration on study medication, compared with 28 of 30 (93%) patients randomized to paltusotine. However, a linear regression model excluding symptom data following rescue did indicate a statistically significant ASEF difference between groups. Although fewer ASD questionnaires were collected at screening (during depot SRL treatment), ASD completion rates during the screening period were similar to those throughout the investigational treatment period of the study. Furthermore, as noted, the symptom exacerbation rate measured during the screening period of this study (30.2% of days [2.1 days per week]) was similar to that measured throughout a 90-day survey period in a separate study of outpatients treated with depot SRLs (32.1% of days [2.2 days per week]; see companion article: Geer EB, et al. “Symptom Severity and Exacerbation Frequency in Medically Treated Patients With Acromegaly”).

The findings from the present analysis are of relevance to the practice of endocrinology. Although measures of disease activity with respect to biochemical control and stability of residual pituitary tumor size are well defined, there is no accepted standard measure of symptom severity or symptom variability. The use of a brief, fit-for-purpose, daily symptom assessment tool enables quantitative measurement of day-to-day variability, as well as average symptom severity. Although useful in a clinical trial, long durations of daily symptom diary-keeping would likely not be needed, even for patients who report breakthrough symptoms in a routine clinical setting. Given the fact that real-world symptom exacerbation rates for patients using depot SRLs were, on average, greater than twice per week (see companion article: Geer EB, et al. “Symptom Severity and Exacerbation Frequency in Medically Treated Patients With Acromegaly”), it should be possible to rule out this degree of breakthrough symptom burden with 1 to 2 weeks of daily symptom assessments prior to maintenance clinic visits.

In conclusion, these results show that patients treated with depot SRLs experience frequent acromegaly symptom exacerbations, even when IGF-I is normal and average symptom severity is controlled. Switching to paltusotine was associated with stable biochemical and symptom severity control and a significantly reduced frequency of symptom exacerbations. A simple, disease-specific, daily symptom assessment tool provided important information pertaining to acromegaly disease control that was not apparent from IGF-I measurements alone. Incorporation of a standard symptom tool into clinical practice, in addition to biochemical and pituitary tumor surveillance, should be considered so as to confirm overall disease control in medically treated patients with acromegaly.

## Supplementary Information

Below is the link to the electronic supplementary material.

Supplementary Material 1

## Data Availability

The datasets used and/or analyzed during the current study are available from the corresponding author upon reasonable request.

## References

[1] Giustina A, Colao A (2025) Acromegaly. N Engl J Med 393(19):1926–1939. 10.1056/NEJMra2409076 41223366

[2] Fleseriu M, Langlois F, Lim DST, Varlamov EV, Melmed S (2022) Acromegaly: pathogenesis, diagnosis, and management. Lancet Diabetes Endocrinol 10(11):804–826. 10.1016/S2213-8587(22)00244-3 36209758

[3] Melmed S, di Filippo L, Fleseriu M, Mercado M, Karavitaki N, Gurnell M, Salvatori R, Tsagarakis S, Losa M, Maffei P, Pereira AM, Geer EB, Katznelson L, van der Lely AJ, Bollerslev J, Esposito D, Webb SM, Zatelli MC, Valassi E, Neggers S, Chanson P, Ho KKY, Ioachimescu AG, Biller BMK, Samson SL, Kaiser UB, Schilbach K, Luque RM, Casanueva FF, Shimon I, Boguszewski CL, Biermasz N, Colao A, Pirchio R, Lamberts SWJ, Kadioglu P, Buchfelder M, Frara S, Chiloiro S, Petersenn S, Gadelha MR, Puig-Domingo M, Luger A, Brue T, Beckers A, Ferone D, Clemmons DR, Greenman Y, Marazuela M, Mortini P, Strasburger CJ, Giustina A (2025) Consensus on acromegaly therapeutic outcomes: an update. Nat Rev Endocrinol 21(11):718–737. 10.1038/s41574-025-01148-2 40804505

[4] Giustina A, Barkhoudarian G, Beckers A, Ben-Shlomo A, Biermasz N, Biller B, Boguszewski C, Bolanowski M, Bollerslev J, Bonert V, Bronstein MD, Buchfelder M, Casanueva F, Chanson P, Clemmons D, Fleseriu M, Formenti AM, Freda P, Gadelha M, Geer E, Gurnell M, Heaney AP, Ho KKY, Ioachimescu AG, Lamberts S, Laws E, Losa M, Maffei P, Mamelak A, Mercado M, Molitch M, Mortini P, Pereira AM, Petersenn S, Post K, Puig-Domingo M, Salvatori R, Samson SL, Shimon I, Strasburger C, Swearingen B, Trainer P, Vance ML, Wass J, Wierman ME, Yuen KCJ, Zatelli MC, Melmed S (2020) Multidisciplinary management of acromegaly: a consensus. Rev Endocr Metab Disord 21(4):667–678. 10.1007/s11154-020-09588-z 32914330 PMC7942783

[5] Broersen LHA, Zamanipoor Najafabadi AH, Pereira AM, Dekkers OM, van Furth WR, Biermasz NR (2021) Improvement in symptoms and health-related quality of life in acromegaly patients: a systematic review and meta-analysis. J Clin Endocrinol Metab 106(2):577–587. 10.1210/clinem/dgaa868 33245343 PMC7823264

[6] Strasburger CJ, Karavitaki N, Störmann S, Trainer PJ, Kreitschmann-Andermahr I, Droste M, Korbonits M, Feldmann B, Zopf K, Sanderson VF, Schwicker D, Gelbaum D, Haviv A, Bidlingmaier M, Biermasz NR (2016) Patient-reported outcomes of parenteral somatostatin analogue injections in 195 patients with acromegaly. Eur J Endocrinol 174(3):355–362. 10.1530/EJE-15-1042 26744896 PMC4722610

[7] Geer EB, Sisco J, Adelman DT, Ludlam WH, Haviv A, Liu S, Mathias SD, Gelbaum D, Shi L (2020) Patient reported outcome data from acromegaly patients treated with injectable somatostatin receptor ligands (SRLs) in routine clinical practice. BMC Endocr Disord 20(1):117. 10.1186/s12902-020-00595-4 32736547 PMC7393879

[8] Fleseriu M, Molitch M, Dreval A, Biermasz NR, Gordon MB, Crosby RD, Ludlam WH, Haviv A, Gilgun-Sherki Y, Mathias SD (2021) Disease and treatment-related burden in patients with acromegaly who are biochemically controlled on injectable somatostatin receptor ligands. Front Endocrinol (Lausanne) 12:627711. 10.3389/fendo.2021.627711 33790860 PMC8006928

[9] Maione L, Albrici C, Grunenwald S, Mouly C, Cimino V, Lecoq AL, Souberbielle JC, Caron P, Chanson P (2022) IGF-I variability over repeated measures in patients with acromegaly under long-acting somatostatin receptor ligands. J Clin Endocrinol Metab 107(9):e3644–e3653. 10.1210/clinem/dgac385 35772775

[10] Remba-Shapiro I, Schweizer JROL, Moscona-Nissan A, Liebert KJP, Jones PS, Schilbach K, Rojas JN, Adams M, Cheng VO, Tritos NA, Bidlingmaier M, Nachtigall LB (2025) Insulin-like growth factor-I and symptoms of acromegaly according to time since somatostatin receptor ligand injection. Eur J Endocrinol 193(4):564–573. 10.1093/ejendo/lvaf200 41001687

[11] Zhao J, Wang S, Markison S, Kim SH, Han S, Chen M, Kusnetzow AK, Rico-Bautista E, Johns M, Luo R, Struthers RS, Madan A, Zhu Y, Betz SF (2023) Discovery of paltusotine (CRN00808), a potent, selective, and orally bioavailable non-peptide SST2 agonist. ACS Med Chem Lett 14(1):66–74. 10.1021/acsmedchemlett.2c00431 36655128 PMC9841592

[12] Madan A, Markison S, Betz SF, Krasner A, Luo R, Jochelson T, Lickliter J, Struthers RS (2022) Paltusotine, a novel oral once-daily nonpeptide SST2 receptor agonist, suppresses GH and IGF-1 in healthy volunteers. Pituitary 25(2):328–339. 10.1007/s11102-021-01201-z 35000098 PMC8894159

[13] Luo R, Madan A, Ferrara-Cook CT, Dalvie D, Goulet L, Struthers RS, Krasner AS (2025) Oral paltusotine, a nonpeptide selective somatostatin receptor 2 agonist: mass balance, absolute bioavailability and metabolism in healthy participants. Br J Clin Pharmacol 91(7):2070–2079. 10.1002/bcp.70020 40040531 PMC12199097

[14] Palsonify (paltusotine) tablets, for oral use [package insert]. Crinetics Pharmaceuticals, Inc. (2025)

[15] Palsonify tablets (2026) SmPc [Summary of Product Characteristics]. Crinetics Pharmaceuticals Europe GmbH

[16] Martin S, Bender RH, Krasner A, Marmon T, Monahan M, Nelson L (2023) Development and evaluation of the Acromegaly Symptom Diary. J Patient Rep Outcomes 7(1):15. 10.1186/s41687-023-00541-7 36792844 PMC9931976

[17] Gadelha MR, Casagrande A, Strasburger CJ, Bidlingmaier M, Snyder PJ, Guitelman MA, Boguszewski CL, Buchfelder M, Shimon I, Raverot G, Tóth M, Mezősi E, Doknic M, Fan X, Clemmons D, Trainer PJ, Struthers RS, Krasner A, Biller BMK (2025) Acromegaly disease control maintained after switching from injected somatostatin receptor ligands to oral paltusotine. J Clin Endocrinol Metab 110(1):228–237. 10.1210/clinem/dgae385 PMC11651685 38828555

[18] Boyd AE, DeFord LL, Mares JE, Leary CC, Garris JL, Dagohoy CG, Boving VG, Brook JP, Phan A, Yao JC (2013) Improving the success rate of gluteal intramuscular injections. Pancreas 42(5):878–882. 10.1097/MPA.0b013e318279d552 23508015

[19] Krishnan T, Safro M, Furlanetto DM, Gill S, Solar Vasconcelos JP, Stuart HC, Martineau P, Loree JM (2024) Clinical impact of unsuccessful subcutaneous administration of octreotide LAR instead of intramuscular administration in patients with metastatic gastroenteropancreatic neuroendocrine tumors. J Neuroendocrinol 36(1):e13360. 10.1111/jne.13360 38088132

[20] Debono M, Hon LQ, Bax N, Blakeborough A, Newell-Price J (2008) Gluteal nodules in patients with metastatic midgut carcinoid disease treated with depot somatostatin analogs. J Clin Endocrinol Metab 93(5):1860–1864. 10.1210/jc.2008-0019 18303072

[21] Sisco J, Palaty C Voice of the patient: living with acromegaly. Paper presented at the Acromegaly Community Virtual Externally-Led Patient-Focused Drug Development Meeting, Virtual, January 21, 2021

[22] Levy MJ (2011) The association of pituitary tumors and headache. Curr Neurol Neurosci Rep 11(2):164–170. 10.1007/s11910-010-0166-7 21128024

[23] Kaniuka-Jakubowska S, Levy MJ, Pal A, Abeyaratne D, Drake WM, Kyriakakis N, Murray RD, Orme SM, Gohil S, Brooke A, Leese GP, Korbonits M, Wass JA (2023) A study of acromegaly-associated headache with somatostatin analgesia. Endocr Relat Cancer 30(3):e220138 . 10.1530/ERC-22-0138 36633458

[24] Puder JJ, Nilavar S, Post KD, Freda PU (2005) Relationship between disease-related morbidity and biochemical markers of activity in patients with acromegaly. J Clin Endocrinol Metab 90(4):1972–1978. 10.1210/jc.2004-2009 15634715

[25] Caron PJ, Bevan JS, Petersenn S, Houchard A, Sert C, Webb SM, PRIMARYS Investigators Group (2016) Effects of lanreotide Autogel primary therapy on symptoms and quality-of-life in acromegaly: data from the PRIMARYS study. Pituitary 19(2):149–157. 10.1007/s11102-015-0693-y 26603536 PMC4799252

[26] Geer EB, Sisco J, Adelman DT, Ludlam WH, Haviv A, Gelbaum D, Liu S, Mathias SD, Shi L (2020) Observed discordance between outcomes reported by acromegaly patients and their treating endocrinology medical provider. Pituitary 23(2):140–148. 10.1007/s11102-019-01013-2 31808101 PMC7066283

