# Supplementary material for: Reduced breakthrough symptom exacerbations in patients with biochemically controlled acromegaly switched from injected depot somatostatin receptor ligands to once-daily oral paltusotine in the PATHFNDR-1 clinical trial
Source: Pituitary. 2026 Jul 23;29(4):128. doi: 10.1007/s11102-026-01733-2 (PMC13395836; doi:10.1007/s11102-026-01733-2)
Supplement: Supplementary file 1 — Supplementary Material 1 [file 11102_2026_1733_MOESM1_ESM.pdf]

Online Resource

Reduced Breakthrough Symptom Exacerbations in Patients With Biochemically Controlled Acromegaly Switched From Injected Depot Somatostatin Receptor Ligands to Once-Daily Oral Paltusotine in the PATHFNDR-1 Clinical Trial

Pituitary

David R. Clemmons, MD; Tiffany P. Quock, PhD, MS; Alessandra Casagrande, MD, PhD; Yang Wang, PhD; Alan Krasner, MD

Corresponding author:
Alan Krasner, MD
Crinetics Pharmaceuticals, Inc.
akrasner@crinetics.com

Suppl. Fig. 1. PATHFNDR-1 study design

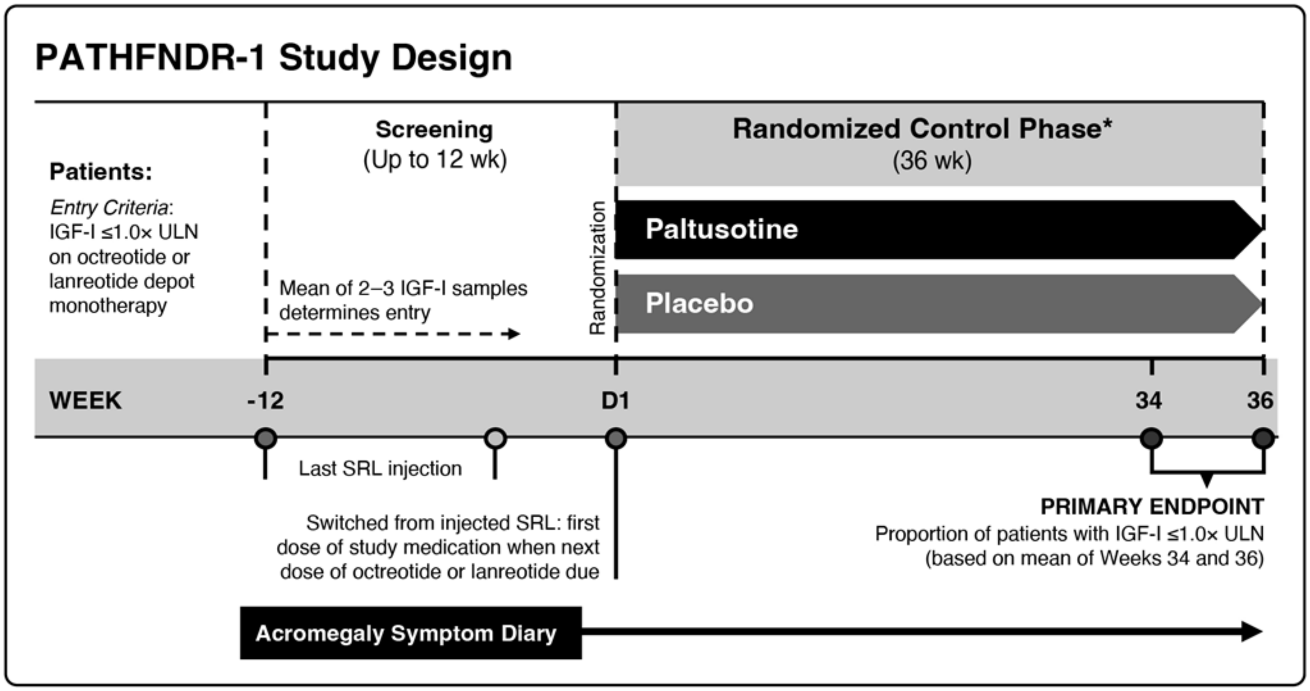

*Per protocol, rescue medication (patient's prior injectable SRL) was administered if there were 2 consecutive IGF-I levels ≥1.3× ULN at the highest dose of study medication (60 mg/day) and exacerbation of acromegaly clinical signs and symptoms as assessed by the investigator.

D, day; IGF-I, insulin-like growth factor 1; SRL, somatostatin receptor ligand; ULN, upper limit of normal.

Figure adapted with permission from Gadelha MR, Casagrande A, Strasburger CJ, Bidlingmaier M, Snyder PJ, Guitelman MA, Boguszewski CL, Buchfelder M, Shimon I, Raverot G, Tóth M, Mezősi E, Doknic M, Fan X, Clemmons D, Trainer PJ, Struthers RS, Krasner A, Biller BMK

(2024) Acromegaly disease control maintained after switching from injected somatostatin receptor ligands to oral paltusotine. J Clin Endocrinol Metab 110(1):228-237, doi:10.1210/clinem/dgae385, via a Creative Commons CC-BY Attribution 4.0 International license (https://creativecommons.org/licenses/by/4.0/), with additional data from the paper.
